# Genetic Variants Related to Cardiometabolic Traits Are Associated to B Cell Function, Insulin Resistance, and Diabetes Among AmeriCan Indians: The Strong Heart Family Study

**DOI:** 10.3389/fgene.2018.00466

**Published:** 2018-10-12

**Authors:** Poojitha Balakrishnan, Dhananjay Vaidya, V. Saroja Voruganti, Karin Haack, Jack W. Kent, Kari E. North, Sandra Laston, Barbara V. Howard, Jason G. Umans, Elisa T. Lee, Lyle G. Best, Jean W. MacCluer, Shelley A. Cole, Ana Navas-Acien, Nora Franceschini

**Affiliations:** ^1^Department of Environmental Health Sciences, Columbia University Mailman School of Public Health, New York, NY, United States; ^2^Department of Epidemiology, Johns Hopkins University Bloomberg School of Public Health, Baltimore, MD, United States; ^3^Clinical and Translational Research, Johns Hopkins School of Medicine, Baltimore, MD, United States; ^4^Department of Nutrition, UNC Nutrition Research Institute, University of North Carolina at Chapel Hill, Kannapolis, NC, United States; ^5^Department of Genetics, Texas Biomedical Research Institute, San Antonio, TX, United States; ^6^Department of Epidemiology, University of North Carolina at Chapel Hill, Chapel Hill, NC, United States; ^7^South Texas Diabetes and Obesity Institute, University of Texas Rio Grande Valley School of Medicine, Brownsville, TX, United States; ^8^MedStar Health Research Institute, Hyattsville, MD, United States; ^9^Georgetown and Howard Universities Center for Clinical and Translational Science, Washington, DC, United States; ^10^Center for American Indian Health Research, College of Public Health, University of Oklahoma Health Sciences Center, Oklahoma City, OK, United States; ^11^Missouri Breaks Industries Research, Inc., Eagle Butte, SD, United States

**Keywords:** American Indian, insulin-secreting cells, insulin resistance, genome-wide association study, diabetes mellitus

## Abstract

**Background:** Genetic research may inform underlying mechanisms for disparities in the burden of type 2 diabetes mellitus among American Indians. Our objective was to assess the association of genetic variants in cardiometabolic candidate genes with B cell dysfunction via HOMA-B, insulin resistance via HOMA-IR, and type 2 diabetes mellitus in the Strong Heart Family Study (SHFS).

**Methods and Results:** We examined the association of variants, previously associated with cardiometabolic traits (∼200,000 from Illumina Cardio MetaboChip), using mixed models of HOMA-B residuals corrected for HOMA-IR (cHOMA-B), log transformed HOMA-IR, and incident diabetes, adjusted for age, sex, population stratification, and familial relatedness. Center-specific estimates were combined using fixed effect meta-analyses. We used Bonferroni correction to account for multiple testing (*P* < 4.13 × 10^−7^). We also assessed the association between variants in candidate diabetes genes with these metabolic traits. We explored the top SNPs in an independent, replication sample from Southwestern Arizona. We identified significant associations with cHOMA-B for common variants at 26 loci of which 8 were novel (*PRSS7, FCRL5, PEL1, LRP12, IGLL1, ARHGEF10, PARVA, FLJ16686*). The most significant variant association with cHOMA-B was observed on chromosome 5 for an intergenic variant near *PARP8* (rs2961831, *P* = 6.39 × 10^−9^). In the replication study, we found a signal at rs4607517 near *GCK/YKT6* (*P* = 0.01). Variants near candidate diabetes genes (especially *GCK* and *KCNQ1*) were also nominally associated with HOMA-IR and cHOMA-B.

**Conclusion:** We identified variants at novel loci and confirmed those at known candidate diabetes loci associations for cHOMA-B. This study also provided evidence for association of variants at *KCNQ2*, *CTNAA2*, and *KCNQ1*with cHOMA-B among American Indians. Further studies are needed to account for the high heritability of diabetes among the American Indian participants of the SHFS cohort.

## Introduction

The burden of type 2 diabetes mellitus among American Indians is disproportionately high with an estimated prevalence ranging between 34 and 68% compared to 9.3% in the general U.S. population (Acton et al., 2002; Lee et al., 2002). In addition to high prevalence, incidence is twice as high among American Indians compared to the U.S. general population (Welty et al., 2002; Narayan et al., 2006). While largely attributed to the obesity epidemic, other risk factors especially among American Indians are not well understood, particularly genetic susceptibility (North et al., 2003; Franceschini et al., 2008; Haiman et al., 2012; Yang et al., 2012).

Pathogenesis is increasingly being attributed to B cell dysfunction compared to insulin resistance in peripheral tissues (Patti, 2004). Heritability for HOMA-beta cell function (HOMA-B), HOMA-IR, type 2 diabetes mellitus are 0.28–0.78, 0.08–0.75, and 0.26–0.70, respectively (Jenkins et al., 2000; Mills et al., 2004; Poulsen et al., 2005; Almgren et al., 2011). Several of the diabetes-associated genes, including *PPARG* and *SLC30A8*, initially identified in individuals of European ancestry, have also been replicated in other populations (Lewis et al., 2008; Chauhan et al., 2010; Yang et al., 2010). Generalization to other ethnic groups has been limited, especially among American Indians (Franceschini et al., 2008; Fesinmeyer et al., 2013; Hanson et al., 2014). Genome-wide linkage analysis in Strong Heart Family Study (SHFS) has demonstrated segregation of diabetes and metabolic trait related variants among American Indian families (North et al., 2003; Franceschini et al., 2008). Yet, most genetic loci identified in other ancestries have not replicated in American Indians (Kovacs et al., 2003; Rong et al., 2009; Haiman et al., 2012; Fesinmeyer et al., 2013). The main goal of this study was to assess the associations between genetic variants previously associated with cardiometabolic traits and dysglycemia traits of HOMA-B, HOMA-IR, and incident diabetes in American Indians among SHFS participants who were free of diabetes at baseline and replicated significant associations in an independent sample from Southwestern Arizona.

## Materials and Methods

### Study Population

The SHFS is an extension of a large, population-based cohort of American Indians in the Strong Heart Study (SHS), recruited from thirteen tribes from three centers: Arizona, Oklahoma and North and South Dakota. Details on participant recruitment and information obtained in clinical visits have been published (Lee et al., 1990; North et al., 2002). Briefly, the SHFS included family members with a core sibship including at least 5 living members of whom at least 3 had to be original SHS participants. Recruitment of the SHFS participants was conducted in two phases; 533 participants attended the baseline visit in 1998–99 and 1,941 participants attended the baseline visit in 2001–03. Follow-up examinations were performed in 2001–03, 2005–06, and 2014–15. Demographic and clinical characteristics were collected at baseline and follow-up visits, including fasting plasma glucose (FPG) and HbA1c. For this study, we included participants without diabetes at baseline since our primary outcome was HOMA-B. We further excluded individuals without measured phenotypic and genotypic data (*n* = 1,923). During follow-up, 256 participants developed diabetes. The replication sample included 3,244 participants from a community in Southwestern Arizona. Detailed information on participant recruitment and data have been described elsewhere (Knowler et al., 1990; Hanson et al., 2013).

All participants in this study provided written informed consent and tribal consent. The study protocols were approved by the Indian Health Service Institutional Review Board, by the Institutional Review Boards of the participating Institutions, and by the participating American Indian tribes.

### Outcome Definition

HOMA-IR (mmol/L) was calculated by fasting insulin in mU/L ^∗^ fasting glucose in mmol/L)/22.5 (Matthews et al., 1985). HOMA-B was calculated using baseline data of participants without diabetes, and the equation described by Matthews et al. (1985) (20 ^∗^ fasting insulin in mU/L)/(fasting glucose in mmol/L - 3.5). To incorporate the influence of insulin resistance, corrected HOMA-B (cHOMA-B) was created as the residuals when HOMA-B was regressed on HOMA-IR (HOMA-B = mean HOMA-B + beta coefficient ^∗^ HOMA-IR). Diabetes was defined as a fasting plasma glucose ≥ 6.99 mmol/L or use of insulin or oral hypoglycemic medications. Incident diabetes was defined as new cases during subsequent follow-up visits (mean follow-up 6.6 years, range 3.0–12.3 years).

### Genotyping

Blood DNA from baseline was genotyped using the Illumina Cardio-Metabo DNA Analysis BeadChip (MetaboChip) (Voight et al., 2010). MetaboChip included 196,725 single nucleotide polymorphisms (SNPs) selected based on meta-analysis of cardiometabolic traits and includes replication targets and fine-mapping regions. Samples were excluded when sample call rate < 95%, mismatch between genotyped and reported gender, outlier in identity by descent (IBD) clustering, or outlier in principal components analysis (PCA). SNPs were excluded if: call rate < 98% or no data (*n* = 33,604); not autosomal (*n* = 250); monomorphic (*n* = 158); or violated Hardy-Weinberg equilibrium (HWE) *P* < 1 × 10^−5^ (*n* = 1,519). PCA was performed on a matrix of doses of copies of minor allele for SNPs selected among genotyped founders or unrelated individuals based on minimum spacing of 1 kb, minor allele frequency (MAF) ≥ 0.05, pairwise correlation of genotype scores < 0.1, and within a sliding window of 100 kb. The first four principal components (PCs) account for substantially more than the rest (cumulatively 8.8% of total variance) (Burdick et al., 2006). In SHFS, the first three PCs cluster by study center and some clustering with less clear separation is apparent for the fourth PC reported in the electronic **Supplementary Material**. Family-based imputation of genotyped SNPs was done with a PEDSYS-compatible version of Merlin using human genome build 18 (NCBI36/hg18); genotype information from relatives was used to impute missing values (Dyke, 1996; Pruitt et al., 2006). After imputation and quality control, 120,975 SNPs were available for analyses.

### Statistical Analysis

All analyses were performed using mixed effects models, to account for family relatedness, for quantitative traits (i.e., HOMA-IR and HOMA-B) and qualitative outcomes (incident diabetes). Analyses was implemented using Sequential Oligogenic Linkage Analysis Routines (SOLAR) assuming additive effect (Blangero and Almasy, 1996). HOMA-B values cannot be evaluated without taking HOMA-IR into account (Pfützner et al., 2010). Based on previous literature, we regressed HOMA-B on HOMA-IR and added the mean HOMA-B to the model residual for interpretability (Willett and Stampfer, 1986; Balakrishnan et al., 2018). These scores were called cHOMA-B and were used as traits in genetic analyses (**Supplementary Figure 1**). Since HOMA-IR values were right-skewed, they were natural log transformed. For HOMA scores, models were adjusted for age at baseline, sex, and first four PCs to adjust for global population stratification. For diabetes, models were adjusted for age at follow-up (mean 42.4 years), sex, and first four PCs. Healthy controls were defined as participants who did had normal or impaired fasting glucose and who were not taking medications. Due to possible differences in allele frequencies among recruiting centers, all models were stratified by recruiting centers. The results from center-stratified association analyses were meta-analyzed using inverse-variance-weighting models implemented using METAL software (Willer et al., 2010).

The array wide significance threshold for multiple testing using Bonferroni correction was 4.13 × 10^−7^ and using the Moskvina and Schmidt method accounting for linkage disequilibrium (LD) was 7.77 × 10^−7^. We performed conditional analysis on the SNPs with lowest *p*-values to identify independent associations at each locus. Models were assessed for genomic inflation (**Supplementary Figure 2**). As sensitivity analyses, we excluded participants with diabetes during follow-up for the HOMA score models. For incident diabetes, we also modified the case definition to include HbA1c thresholds and control definition to exclude participants with impaired fasting glucose. Replication of significant SNPs was assessed for nominal significance (*P* < 0.05) in an independent sample from a community in Southwestern Arizona (*n* = 3,244) without diabetes for cHOMA-B. Models were adjusted for age, sex, and first five PCs to account for population stratification in the sample.

## Results

**Table 1** shows the baseline characteristics of the study population. Over a mean follow-up of 6.6 years (12,667.6 person-years), 256 participants or 13.5% of the study population developed incident diabetes. On average, participants who developed diabetes had nearly 1 year longer follow-up than participants who did not develop diabetes. Participants developing incident diabetes were older, more often obese, and had a higher fasting glucose, HOMA-B and HOMA-IR at baseline (*P* < 0.05). When corrected for HOMA-IR, cHOMA-B was lower among those who developed diabetes (median 158.1) compared to those who did not develop diabetes during follow-up (median 188.6) (*P* < 0.01).

**Table 1:** Baseline characteristics by development of diabetes during follow-up.

	Total	Incident diabetes	Controls^b^	*P*-value
Sample size	1,892	256	1,636	
Median follow-up, yrs (IQR)	6.1 (5.0–8.0)	6.9 (5.5–9.6)	6.0 (5.0–7.6)	<0.01
Mean age, years (SD)	36.4 (15.5)	39.2 (14.1)	36.0 (15.7)	<0.01
No. Female (%)	1,140 (60.3)	146 (57.0)	994 (60.8)	0.26
Center (%)				<0.01
Arizona	195 (10.3)	48 (18.8)	147 (9.0)	
Oklahoma	796 (42.1)	92 (35.9)	704 (43.0)	
North/South Dakota	901 (47.6)	116 (45.3)	785 (48.0)	
Mean BMI, kg/m2 (SD)	30.4 (7.3)	35.7 (8.5)	29.6 (6.7)	<0.01
Mean fasting glucose, mmol/L (SD)	5.2 (0.6)	5.6 (0.6)	5.1 (0.5)	<0.01
Mean fasting insulin, pmol/L (SD)	115.3 (114.6)	182.7 (150.0)	104.9 (104.2)	<0.01
Median HOMA-IR (IQR)	2.7 (1.7–4.6)	5.0 (3.3–8.3)	2.5 (1.6–4.1)	<0.01
Median HOMA-B (IQR)	155.2 (104.2–246.0)	201.0 (134.1–335.8)	148.7 (99.5–231.3)	<0.01
Median cHOMA-B^a^ (IQR)	186.1 (153.3–235.9)	158.1 (109.1–219.1)	188.6 (157.4–237.3)	<0.01

*^a^Corrected HOMA-B; Sum of residuals adjusted for HOMA-IR and mean HOMA-B. ^b^Controls are individuals that did not develop diabetes at follow-up.*

**FIGURE 1:**
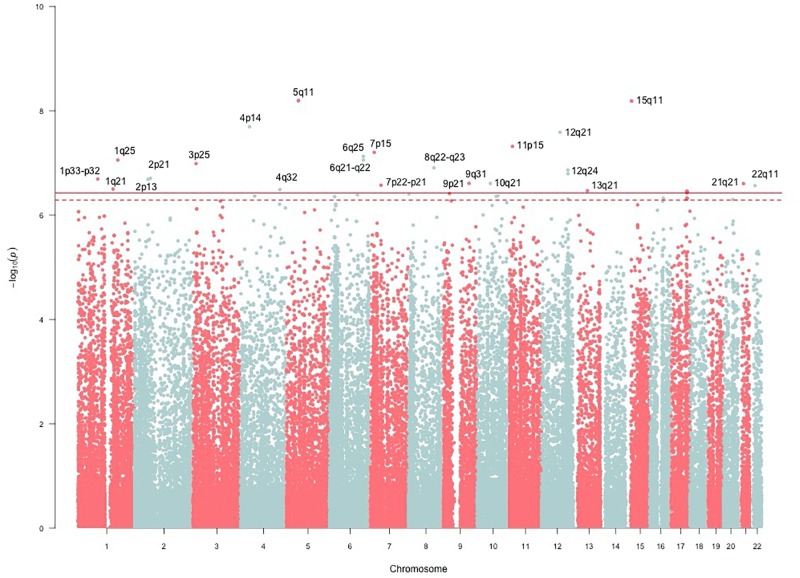
Manhattan plot of corrected HOMA-B. SNP associations mapped using NCBI36/hg18 build position. The solid line is the MetaboChip-wide Bonferroni significance threshold at −log(4.13 × 10^−7^) or 6.38. The dashed line is the MetaboChip-wide Moskvina-Schmidt LD significance threshold at −log(7.77 × 10^−7^) or 6.11.

**FIGURE 2:**
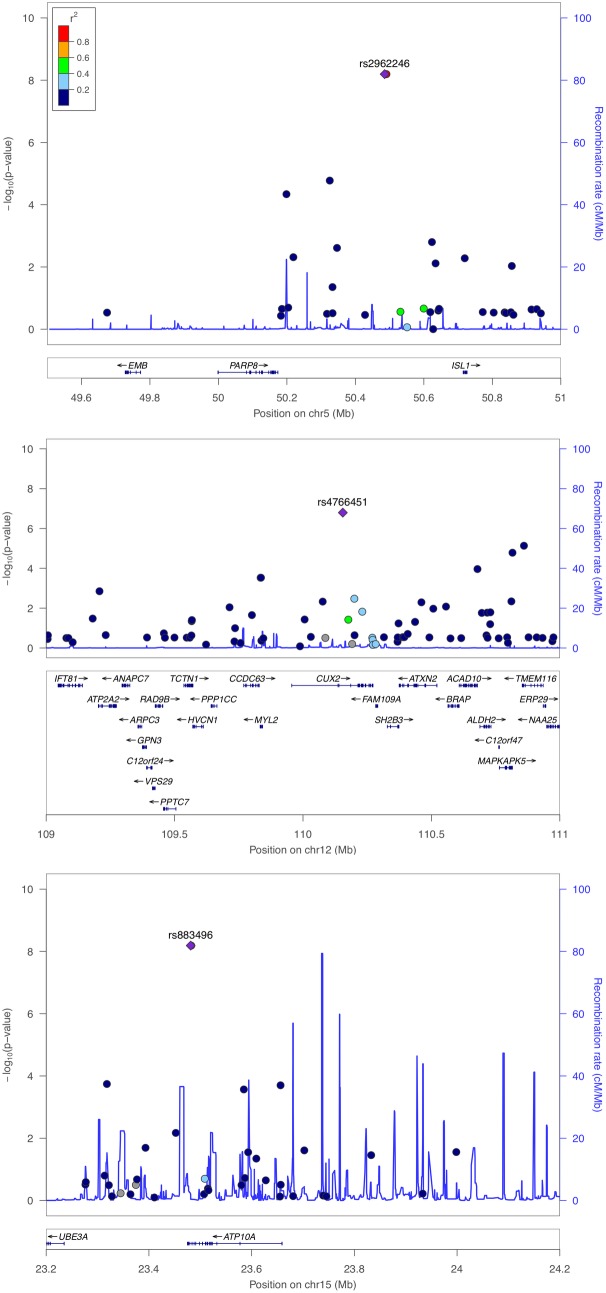
Regional association plot of PARP8, CUX2, and ATP10A for corrected HOMA-B. Multiple SNP associations for cHOMA-B are 5q11/PARP8 (top panel), 12q24/CUX2 (middle panel), and 15q12/ATP10A (bottom panel), using NCBI36/hg18 build and European American recombination rates.

The genomic inflation factor (λ) for cHOMA-B, HOMA-IR, and diabetes were 1.11, 1.02, and 1.24, respectively (**Supplementary Figure 2**). We identified several genes in the Cardio MetaboChip significantly associated with cHOMA-B (*P* < 4.13 × 10^−7^) (**Supplementary Table 1**). A total of 28 variants in 25 distinct loci were statistically significantly associated with cHOMA-B (**Figure 1**). The associations observed near *PRSS7*, *FCRL5*, *PEL1*, *LRP12*, *IGLL1*, *ARHGEF10*, *PARVA*, and *FLJ16686* are novel for a biomarker of B cell dysfunction. *GCK* demonstrated the most consistent association with B-cell function phenotypes. In particular, rs4607517 (G > A) was associated with a decrease in cHOMA-B of 19.54 units in SHFS (*P* = 2.67 × 10^−7^) and 11.26 in the replication sample (*P* = 0.01). The most significant SNP associated with cHOMA-B was rs2961831 (A > C), an intergenic variant, located at 5q11/*PARP8* (**Table 2**). Each copy of allele C was associated with increases of 23.11 units of cHOMA-B (*P* = 6.39 × 10^−9^). Of the variants, 3 regions at 5q11/*PARP8*, 12q24/*CUX2*, and 15q12/*ATP10A* had multiple variant associations that passed the significance threshold (**Figure 2**). Conditional analysis on index SNPs suggested the presence of a single association at each locus. Results for all significant SNP associations for log transformed HOMA-B and cHOMA-B are presented as **Supplementary Material** (**Supplementary Table 1**).

**Table 2:** Main associations for cHOMA-B and log HOMA-IR.

rsID	Chr	Position	Alleles	Gene	Location	Strong heart family study			Replication sample		
						MAF	Beta	*P*-value	MAF	Beta	*P*-value
rs284201	1	92012826	G/A	*TGFBR3*	intron	0.27	16.72	*2.04 × 10*^−^*^7^*	0.19	−1.75	0.72
rs1972239	1	155775425	A/G	*FCRL5*	intron	0.18	22.00	*3.17 × 10*^−^*^7^*	0.15	−6.06	0.25
rs1252068	1	175868691	A/G	*LOC400796, SEC16B*	intergenic	0.35	−18.61	*8.80 × 10*^−^*^8^*	0.45	−2.09	0.58
rs7588499	2	54581780	A/G	*SPTBN1*	intron	0.49	13.76	*2.06 × 10*^−^*^7^*	0.41	−1.13	0.77
rs6748843	2	64383898	A/G	*PELI1, LOC130773*	intergenic	0.47	18.01	*1.97 × 10*^−^*^7^*	0.35	−6.74	0.10
rs3732678	3	12836608	A/C	*CAND2*	coding	0.25	20.22	*1.03 × 10*^−^*^7^*	0.40	−0.10	0.98
rs12642615	4	36914666	G/A	*FLJ16686, KIAA1239*	intergenic	0.38	16.88	*2.02 × 10*^−^*^8^*	0.32	−0.16	0.97
rs4139	4	163610383	A/G	*FSTL5, LOC729971*	intergenic	0.35	16.99	*3.23 × 10*^−^*^7^*	0.35	−3.79	0.35
rs2961831	5	50490690	A/C	*PARP8, LOC642366*	intergenic	0.40	23.11	*6.39 × 10*^−^*^9^*	0.35	0.26	0.95
rs2962246	5	50485571	A/G	*PARP8, LOC642366*	intergenic	0.40	23.11	*6.39 × 10*^−^*^9^*	0.35	0.21	0.96
rs4448128	6	116137499	C/G	*LOC441167, FRK*	intergenic	0.25	−16.62	*4.08 × 10*^−^*^7^*	0.16	−6.92	0.18
rs654993	6	160488886	G/A	*SLC22A1*	intron	0.23	18.31	*7.74 × 10*^−^*^8^*	0.14	1.74	0.75
rs38179	7	15865240	A/G	*MEOX2, LOC729920*	intergenic	0.45	21.54	*6.26 × 10*^−^*^8^*	0.48	3.05	0.42
rs4607517	7	44202193	G/A	*GCK, YKT6*	intergenic	0.26	−19.54	*2.67 × 10*^−^*^7^*	0.31	−11.26	0.01
rs7386942	8	1890157	G/A	*ARHGEF10*	intron	0.48	−14.43	*3.94 × 10*^−^*^7^*	0.50	−7.24	0.05
rs16872183	8	106094163	A/G	*LRP12, ZFPM2*	intergenic	0.26	−22.62	*1.24 × 10*^−^*^7^*	0.37	−5.14	0.19
rs10738708	9	25231316	A/C	*TUSC1, LOC10012966*	intergenic	0.43	17.26	*3.89 × 10*^−^*^7^*	0.40	−1.91	0.61
rs4149270	9	106686898	G/A	*ABCA1*	intron	0.23	−22.99	*2.46 × 10*^−^*^7^*	0.18	−0.50	0.51
rs997067	10	55709300	A/C	*PCDH15*	intron	0.37	20.51	*2.47 × 10*^−^*^7^*	0.29	4.17	0.32
rs4237723	11	12594808	A/G	*PARVA, TEAD1*	intergenic	0.45	18.33	*4.82 × 10*^−^*^8^*	0.40	−3.36	0.38
rs10777559	12	76822590	C/A	*NAV3*	intron	0.25	−26.21	*2.58 × 10*^−^*^8^*	0.17	−1.50	0.77
rs10744770	12	110146961	G/A	*CUX2*	intron	0.21	−23.4	*1.36 × 10*^−^*^7^*	0.16	−3.87	0.45
rs4766451	12	110155065	C/A	*CUX2*	intron	0.22	−23.46	*1.60 × 10*^−^*^7^*	0.17	−3.82	0.45
rs9528062	13	59628504	G/A	*DIAPH3*	intron	0.28	−18.51	*3.40 × 10*^−^*^7^*	0.24	−2.23	0.61
rs883496	15	23481396	G/A	*ATP10A*	intron	0.47	−23.44	*6.49 × 10*^−^*^9^*	0.48	1.01	0.79
rs10152552	15	23482358	A/G	*ATP10A*	intron	0.47	23.44	*6.49 × 10*^−^*^9^*	0.48	1.02	0.79
rs2826602	21	21198206	G/A	*PRSS7, NCAM2*	intergenic	0.34	−18.17	*2.49 × 10*^−^*^7^*	0.39	5.94	0.13
rs131409	22	22221107	A/G	*LOC388882, IGLL1*	intergenic	0.27	19.82	*2.72 × 10*^−^*^7^*	0.32	−3.28	0.42

*Gene base position based on NCBI36/hg18 build. P-values of significant SNPs are italicized (*P* < 4.13 × 10^−7^). Chr, chromosome; cHOMA-B, corrected homeostatic model assessment – B cell function; MAF, minor allele frequency. follow-up.*

We assessed the top 28 SNPs for cHOMA-B in an independent sample from Southwestern Arizona (**Table 2**). Variants had similar MAF in the replication sample compared to the SHFS, but we were not able to replicate many of these associations in the replication sample except rs4607517. One variant showed nominal significance; an intergenic SNP rs4607517 (G > A; MAF in replication sample 0.31) showed an effect estimate of –11.26 (*P* = 0.01) compared to an effect estimate of –19.54 (*P* = 2.67 × 10^−7^) in the SHFS (MAF 0.26).

The top association for log transformed HOMA-IR was with rs7609071 (G > C) near *CTNAA2* (*P* = 1.34 × 10^−5^) (**Supplementary Table 1**). Each copy of the C allele was associated with an increase of 0.84 and 0.79 units of HOMA-IR in Oklahoma, and the Dakotas respectively, (effect estimate –0.18, –0.24) (**Supplementary Table 1**). Other SNPs at 2p11-12 were nominally associated with cHOMA-B. In addition to being associated with cHOMA-B, rs7609071 was associated with the index HOMA-IR (*P* = 2.51 × 10^−3^) but not with incident diabetes (*P* = 0.87). There was no significant evidence for association of SNPs with incident diabetes (**Supplementary Table 1**).

In the MetaboChip data, there were seven candidate diabetes loci (**Supplementary Table 2**) with 1,446 SNPs that passed quality control. Among the observed associations, an intronic SNP rs163170 (*KCNQ1*) was significantly associated with HOMA-IR and with cHOMA-B (**Table 3**). This variant is located near the previously reported SNP at this locus (rs8181588) although their correlation is low in our American Indian population (LD r^2^ = 0.29) (McCarthy and Zeggini, 2009; Hanson et al., 2014). None of the MetaboChip SNPs in the candidate genes were associated with incident diabetes.

**Table 3:** Main candidate gene associations for cHOMA-B and log HOMA-IR.

rsID	Chr	Position	Alleles	MAF	Gene	Location	cHOMA-B		HOMA-IR		Diabetes	
							Beta	*P*-value	Beta	*P*-value	Beta	*P*-value
rs4135273	3	12414329	G/A	0.01	*PPARG*	intron	−0.01	0.01	−0.21	0.05	−0.04	0.62
rs16860216	3	186971576	G/A	0.13	*IGF2BP2*	intergenic	0.13	0.02	−0.07	0.03	−0.03	0.57
rs3731201	9	21978896	A/G	0.04	*CDKN2A*	intron	−0.01	0.09	−0.02	0.39	−0.01	0.76
rs3218018	9	21988139	A/C	0.03	*CDKN2B*	intergenic	−0.06	0.18	−0.08	0.24	−0.05	0.72
rs4639863	10	114844373	G/A	0.37	*TCF7L2*	intergenic	0.23	0.01	0.08	0.04	−0.01	0.75
rs163170	11	2778003	G/A	0.50	*KCNQ1*	intron	0.04	6.27 × 10^−6^	−0.11	2.16 × 10^−4^	−0.01	0.92
rs35379941	12	119909772	T/A	0.11	*HNF1A*	intron	−0.01	0.35	0.06	0.17	0.02	0.41

*Gene base position based on NCBI36/hg18 build. Chr, chromosome; cHOMA-B, corrected homeostatic model assessment – B cell function; MAF, minor allele frequency. P < 0.05 for significant replication.*

## Discussion

Our study validates diabetes-related loci in American Indians, a population with a large burden of diabetes. Among the SHFS participants, the diabetes prevalence was 15.6%, which is high compared to the 9.3% prevalence reported in the general U.S. population (Acton et al., 2002; Lee et al., 2002). The diabetes prevalence is especially disproportionate among younger age groups, where the crude prevalence in NHANES among 18–44 ages was 5.0% (Menke et al., 2015) compared to 12.4% in the SHFS. Our study demonstrated that known cardiometabolic loci are involved in B cell dysfunction and insulin resistance in American Indians. In particular, we identified *GCK* variant rs4607517, which was previously identified including MAGIC consortium and MESA cohort (Dupuis et al., 2010; Rasmussen-Torvik et al., 2012). We also identified some novel associations using the Cardio MetaboChip array, such as the association of cHOMA-B with *PARP8*. In the diabetes candidate gene analysis, we identified an association of an intronic SNP rs163170 in *KCNQ1* with both cHOMA-B and HOMA-IR.

The variants associated with cHOMA-B are located near or in genes that show involvement in biological mechanisms such as metabolism (*GCK*, *PRSS7*), movement across extracellular and intracellular membranes (*FCRL5*, *SEC16B*, *TGFBR3*, *PEL1*, *FSTL5*, *SLC22A1*, *LRP12*, *ABCA1*, *ATP10A*, *KCNJ2*, *NCAM2*, *IGLL1*), activation and deactivation of hydrolase enzymes (*ARHGEF10*, *NAV3*), transcriptional and DNA-binding regulation (*CAND2*, *ZFPM2*, *TUSC1*, *TEAD1*, *CUX2*), cell movement and adhesion (*SPTBN1*, *PCDH15*, *PARVA*, *DIAPH3*), and cell cycle and apoptosis (*FLJ16686*, *FRK*, *MEOX2*) (Pruitt et al., 2006). The most significant variants for cHOMA-B were located at *PARP8* [Poly(adenosine disphosphate/ADP-ribosyl)ation – member VIII], which catalyze the transfer of ADP-ribose from glutamic acid to aspartic acid, possibly involving zinc fingers (Amé et al., 2004). Both *CUX2* (Cut like homeobox 2), a DNA binding motif, and *ATP10A* (ATPase phospholipid transporting 10A), a maternally expressed aminophospholipid translocase across the lipid bilayer, were previously reported in associations with diabetes related traits including metabolic syndrome (Shim et al., 2014) and insulin resistance (Irvin et al., 2011). In this study, we identified associations with variants at loci not encompassed in linkage peaks on chromosomes 3 and 4 as previously reported in the SHFS (North et al., 2005). Our replication yielded one nominal association for rs4607517 near *GCK/YKT6* with consistent directions for the beta estimates. This was the only association that has been previously identified to be associated with HOMA-B (Dupuis et al., 2010). Overall, we did not find consistency between the findings in the two samples possibly due to heterogeneity between the cohorts and centers in SHFS. There may be heterogeneity in LD structure between the populations. A preliminary measure is the notable difference in MAF particularly in the variants of top candidate genes (*GCK* and *KCNQ1*). There may also be heterogeneity in measurement of variables including phenotypes that could partially account for the differences in effect sizes and statistical significance.

In analyses of diabetes candidate genes, we identified an association with several variants near *KCNQ1*, which encodes a voltage-gated potassium channel and has been associated with diabetes in individuals of European and East Asian ancestries but also in Southwest American Indians (Schroeder et al., 2000; McCarthy and Zeggini, 2009). *KCNQ1* variants showed evidence of functional effects in knockout mice (Hanson et al., 2014). *In vitro* and murine studies have shown that the overactivity of the potassium channels from overexpression of *KCNQ1* can create a current across the plasma membrane and impair insulin secretion, thereby resulting in hyperglycemia (McCarthy and Zeggini, 2009; Yamagata et al., 2011). In a study among American Indians from central Arizona, *KCNQ1* variants were associated with incident diabetes (Franceschini et al., 2013; Hanson et al., 2014). In our study, *KCNQ1* variants are related to both a decrease in HOMA-IR (effect -0.11) and with an increase in cHOMA-B (effect 0.04). ENCODE data using HaploReg (v4.0) of the diabetes candidate genes shows possible functional regulation including binding to DNA hypersensitivity site, histone promoter and enhancer sites to investigate in future studies.

Our study is one of the few that have investigated the association of genetic determinants of diabetes traits among American Indians. While the number of participants who developed diabetes was relatively small for a genetic analysis, we still identified several significant associations with cHOMA-B. The small sample size with incident diabetes could partly account for why our study was not able to replicate associations seen in other ethnic groups. The SHFS is a unique cohort especially for genetic studies because of the inclusion of related participants from complex pedigrees with high burden of diabetes. This allows for possible fine-mapping of association signals by exploiting differences in LD patterns between American Indians and European Americans among whom most association studies have been conducted. Moreover, it allows for the examination of generalizability of the detected associations in non-European American ethnic groups. We tested variants from the MetaboChip as it provides good coverage and also prioritizes cardiometabolic SNPs and thus minimizes multiple testing. Although it provides overall good coverage in different populations, the MetaboChip or any GWAS chip for that matter may miss SNPs in American Indian populations and measured SNPs may have lower allele frequencies in American Indian populations. Even so, our study was able to replicate some associations (Kovacs et al., 2003; Rong et al., 2009; Yang et al., 2010). While the top SNPs showed limited evidence of consistency in our replication sample, we believe this warrants further investigation of the heterogeneity of genetic susceptibility among American Indians. We observed inflation in the genomic control factor (λ) statistic. However, previous investigations in other cohorts have also reported this inflation when using the MetaboChip panel, most likely because of fine-mapping of several loci (Huertas-Vazquez et al., 2013). In addition, none of the significant findings reported for cHOMA-B were in the loci showing deviation from the diagonal.

Also, HOMA-B and HOMA-IR are surrogate measures of B cell dysfunction and insulin resistance, respectively, and therefore may not truly reflect mechanisms of developing diabetes (Wallace et al., 2004). There is limited assessment of the validity of HOMA scores, especially among various ethnic groups. Pfützner et al. (2010) conducted a randomized control trial that highlighted the inability of HOMA-B scores to replicate the findings of laboratory measures of B cell function. Yet compared to other measures, HOMA models are favorably used in diabetes epidemiologic due to ease of measure and accuracy (Song et al., 2007). Another caveat for HOMA-B is that it cannot be interpreted without accounting for HOMA-IR. We used methodology developed in nutritional epidemiology to account for correlated measures or as in the case with HOMA-B and HOMA-IR, measures built from similar underlying variables (Willett and Stampfer, 1986). Thus, we corrected for the influence of HOMA-IR on HOMA-B by using the HOMA-B residuals and adding a constant of mean HOMA-B. The cHOMA-B phenotype is also less well studied and therefore makes it difficult to compare with previously reported associations. Finally, we cannot discount the possibility that our incident diabetes participants include type 1 diabetes as is usual in large epidemiologic cohorts such as the SHFS. However, given that participants who developed diabetes during follow-up were middle-aged (mean 39.2 years), we believe the number of participants who may have had type 1 diabetes is low.

The novel and replicated associations for cHOMA-B provide new information of genetic association for diabetes traits in American Indians. We also validated associations of *GCK* and *KCNQ1* variants with diabetes in our study population. Although our study has a small number of incident cases and did not validate diabetes-associated loci, we identified several variants in novel loci that are hypothesis-generating for understanding genetic susceptibility in American Indians. Further studies with larger sample size and dense markers are needed to validate our results and identify additional loci unique to American Indians. Further investigation is therefore warranted to better understand the genetic susceptibility to diabetes among American Indians.

## Data Availability

The datasets for this manuscript are not publicly available because data is governed by the review of the Strong Heart Steering Committee to ensure that the researchers agree that tribes need to review and approve the manuscripts before submission for publication and to ensure that researchers are responsible and respect the ethical concerns and requirements of the American Indian communities. Requests to access the datasets should be directed to Dr. Shelley Cole, scole@txbiomed.org.

## Ethics Statement

This study was carried out in accordance with the recommendations of Indian Health Service Institutional Review Board, by the Institutional Review Boards of the participating Institutions, and by the participating American Indian tribes with written informed consent from all subjects. All subjects gave written informed consent in accordance with the Declaration of Helsinki. The protocol was approved by the Indian Health Service Institutional Review Board, by the Institutional Review Boards of the participating Institutions, and by the participating American Indian tribes.

## Author Contributions

All authors were involved in conception and design of study, drafting and revising of the manuscript, and final approval of paper.

## Conflict of Interest Statement

LB was employed at Missouri Breaks Industries Research, Inc. The remaining authors declare that the research was conducted in the absence of any commercial or financial relationships that could be construed as a potential conflict of interest.

## Abbreviations

cHOMA-B: HOMA-beta cell function residuals corrected for HOMA-IR
chr: chromosome
FPG: fasting plasma glucose
HOMA-B: HOMA-beta cell function
HWE: Hardy-Weinberg equilibrium
IBD: identity by descent
MetaboChip: Illumina Cardio-Metabo DNA Analysis BeadChip
MAF: minor allele frequency
PC: principal component
PCA: principal components analysis
SHFS: Strong Heart Family Study
SHS: Strong Heart Study
SNP: single nucleotide polymorphism
