# Supplementary material for: Genetic Variants Related to Cardiometabolic Traits Are Associated to B Cell Function, Insulin Resistance, and Diabetes Among AmeriCan Indians: The Strong Heart Family Study
Source: Front Genet. 2018 Oct 12;9:466. doi: 10.3389/fgene.2018.00466 (PMC6194194; doi:10.3389/fgene.2018.00466)
Supplement: Supplementary file 1 [file Table_1.pdf]

Supplementary Material

Genetic Variants Related to Cardiometabolic Traits Are Associated to B Cell Function, Insulin Resistance, and Diabetes among American Indians: The Strong Heart Family Study

Poojitha Balakrishnan*, Dhananjay Vaidya, V. Saroja Voruganti, Karin Haack, Jack W. Kent, Jr, Kari E. North, Sandra Laston, Barbara V. Howard, Jason Umans, Elisa T. Lee, Lyle G. Best, Jean W. MacCluer, Shelley A. Cole, Ana Navas-Acien, Nora Franceschini

*** Correspondence:** Poojitha Balakrishnan: pbalakr2@jhu.edu

1 Supplementary Tables

1.1 Supplementary Table 1. SNP Associations for HOMA-B, log HOMA-IR, and incident diabetes.

rsID	Chr	Position	Alleles	MAF	Gene	Locus	Location	Log HOMA-B		Log HOMA-IR		Diabetes	
								Beta	P-value	Beta	P-value	Beta	P-value
rs2961831	5	50490690	A/C	0.40	*PARP8, LOC642366*	5q11	intergenic	0.08	0.05	0.04	0.13	-0.03	0.34
rs2962246	5	50485571	A/G	0.40	*PARP8, LOC642366*	5q11	intergenic	0.08	0.05	0.04	0.13	-0.03	0.34
rs10152552	15	23482358	A/G	0.47	*ATP10A*	15q11	intron	0.08	0.03	0.02	0.46	-0.01	0.71
rs883496	15	23481396	G/A	0.47	*ATP10A*	15q11	intron	-0.08	0.03	-0.02	0.46	0.01	0.71
rs12642615	4	36914666	G/A	0.38	*FLJ16686, KIAA1239*	4p14	intergenic	0.08	0.05	-0.01	0.95	-0.01	0.70
rs10777559	12	76822590	C/A	0.25	*NAV3*	12q21	intron	-0.09	0.04	-0.02	0.46	0.001	0.98
rs4237723	11	12594808	A/G	0.45	*PARVA, TEAD1*	11p1	intergenic	0.03	0.36	-0.02	0.4	-0.01	0.74
rs38179	7	15865240	A/G	0.45	*MEOX2, LOC729920*	7p22-p21	intergenic	0.08	0.03	0.01	0.76	0.01	0.65
rs654993	6	160488886	G/A	0.23	*SLC22A1*	6q25	intron	0.07	0.11	-0.01	0.72	-0.002	0.96
rs1252068	1	175868691	A/G	0.35	*LOC400796, SEC16B*	1q25	intergenic	0.02	0.53	0.05	0.07	-0.007	0.84
rs3732678	3	12836608	A/C	0.25	*CAND2*	3p25	coding	0.15	6.03×10^{-4}	0.08	0.01	0.002	0.97
rs16872183	8	106094163	A/G	0.26	*LRP12, ZFPM2*	8q22-q23	intergenic	-0.10	0.03	-0.05	0.14	0.02	0.55
rs10744770	12	110146961	G/A	0.21	*CUX2*	12q24	intron	-0.12	0.02	-0.03	0.39	0.02	0.62

rs4766451	12	110155065	C/A	0.22	*CUX2*	12q24	intron	-0.11	0.02	-0.02	0.51	0.02	0.54
rs6748843	2	64383898	A/G	0.47	*PELI1, LOC130773*	2p13	intergenic	0.00	0.96	0.01	0.74	0.02	0.60
rs284201	1	92012826	G/A	0.27	*TGFBR3*	1p33-p32	intron	0.03	0.44	0.02	0.55	-0.003	0.93
rs7588499	2	54581780	A/G	0.49	*SPTBN1*	2p21	intron	0.00	0.92	-0.03	0.33	0.01	0.68
rs4149270	9	106686898	G/A	0.23	*ABCA1*	9q31	intron	-0.03	0.50	-0.01	0.64	-0.001	0.98
rs997067	10	55709300	A/C	0.37	*PCDH15*	10q21	intron	-0.03	0.43	-0.05	0.09	-0.01	0.71
rs2826602	21	21198206	G/A	0.34	*PRSS7, NCAM2*	21q21	intergenic	-0.03	0.45	-0.01	0.99	0.004	0.89
rs4607517	7	44202193	G/A	0.26	*GCK, YKT6*	7p15	intergenic	-0.11	0.01	-0.05	0.12	0.003	0.94
rs131409	22	22221107	A/G	0.27	*LOC388882, IGLL1*	22q11	intergenic	0.03	0.46	-0.04	0.23	-0.005	0.86
rs1972239	1	155775425	A/G	0.18	*FCRL5*	1q21	intron	0.17	$5.30x10^{-4}$	0.06	0.09	0.0001	0.99
rs4139	4	163610383	A/G	0.35	*FSTL5, LOC729971*	4q32	intergenic	0.01	0.82	-0.04	0.12	-0.02	0.61
rs9528062	13	59628504	G/A	0.28	*DIAPH3*	13q21	intron	0.01	0.84	0.07	0.01	0.009	0.75
rs312726	17	65799923	T/G	0.34	*KCNJ2, LOC100133226*	17q24	intergenic	0.01	0.78	0.02	0.38	-0.002	0.89
rs420572	17	65808955	T/C	0.34	*KCNJ2, LOC100133226*	17q24	intergenic	0.01	0.76	0.03	0.34	-0.01	0.73
rs312701	17	65809179	G/C	0.34	*KCNJ2, LOC100133226*	17q24	intergenic	0.04	0.32	0.03	0.30	-0.01	0.73
rs10738708	9	25231316	A/C	0.43	*LOC100129669, TUSC1*	9p21	intergenic	0.04	0.32	0.06	0.04	-0.005	0.89
rs7386942	8	1890157	G/A	0.48	*ARHGEF10*	8p23	intron	-0.04	0.30	-0.02	0.46	-0.0004	0.99
rs4448128	6	116137499	C/G	0.25	*LOC441167, FRK*	6q21-q22	intergenic	-0.06	0.14	-0.02	0.43	0.008	0.81

Gene base position based on NCBI36/hg18 build. P-values of significant SNPs are italicized ($P < 4.13x10^{-7}$). Abbreviations: Chr, chromosome; MAF, minor allele frequency.

1.2 Supplementary Table 2. Candidate Diabetes Genes.

Gene	Description	Chr	Position	No. MetaboChip SNPs
PPARG	peroxisome proliferator-activated receptor gamma	3	12,368,001-12,450,855	153
IGF2BP2	insulin-like growth factor 2 mRNA binding	3	186,844,221-187,025,521	212
CDKN2A	cyclin-dependent kinase inhibitor 2A	9	21,957,751-21,965,038	26
CDKN2B	cyclin-dependent kinase inhibitor 2B	9	21,992,902-21,999,312	53
TCF7L2	transcription factor 7-like 2	10	114,699,999-114,916,060	133
KCNQ1	potassium voltage-gated channel, KQT-like	11	2,422,797-2,826,916	836

HNF1A	transcription factor 1, hepatic	12	119,900,932-119,924,697	33

List of SNPs localized to candidate diabetes genes by MetaboChip gene annotation that pass quality control. Gene base position based on NCBI36/hg18 build.

2 Supplementary Figures

2.1 Supplementary Figure 1. Correlation Plots for HOMA-B and corrected HOMA-B.

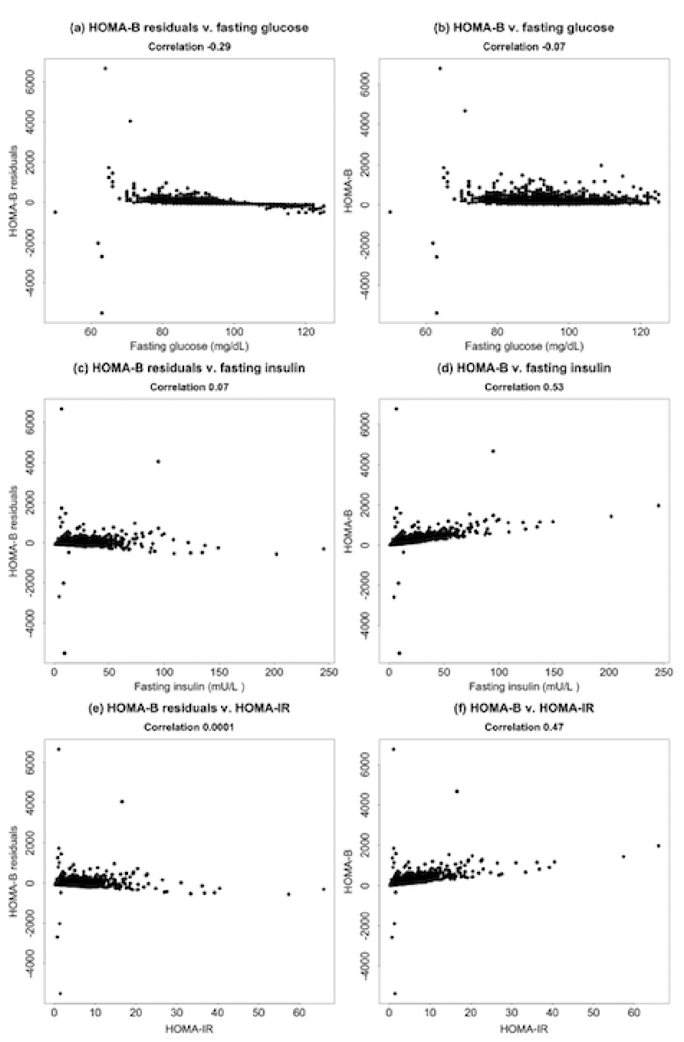

Pearson correlation calculated with fasting glucose, fasting insulin, and HOMA-IR for HOMA-B [(a), (c), (e)] and cHOMA-B [(b), (d), (f)], respectively.

2.2 Supplementary Figure 2. Quantile-Quantile Plots for HOMA-B Residuals, Log HOMA-IR, and Diabetes.

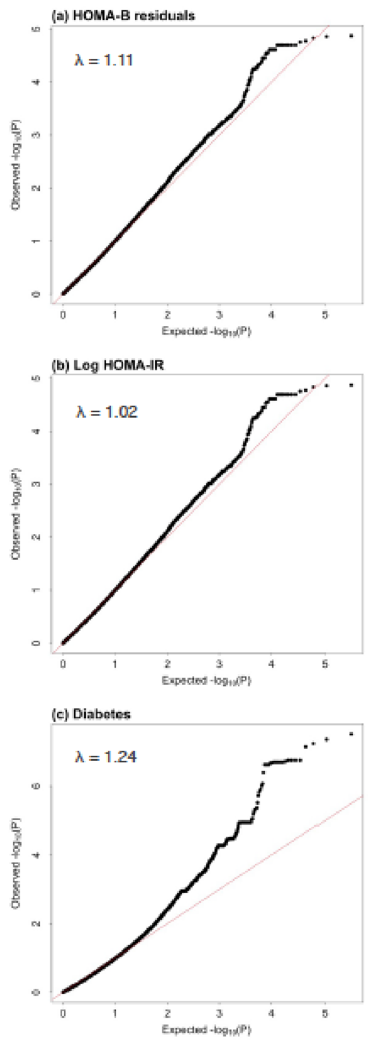

Deviation from expected p-values adjusted for genomic inflation using MetaboChip for (a) HOMA-B residuals, (b) Log HOMA-IR, and (c) Diabetes. The genomic inflation factor (λ) for cHOMA-B, HOMA-IR, and diabetes were estimated to be 1.11, 1.02, and 1.24, respectively
